# Supplementary material for: Content-rich biological network constructed by mining PubMed abstracts
Source: BMC Bioinformatics. 2004 Oct 8;5:147. doi: 10.1186/1471-2105-5-147 (PMC528731; doi:10.1186/1471-2105-5-147)
Supplement: Additional File 2 — The original results of the above study (non-essential files are deleted to keep the file size under the limit set by BMC bioinformatics). [file 1471-2105-5-147-S2.bz2 › chilibotAdditionalFile2/dip20/327ID9546397E3626/html/TFRC_TF.html]

 


 **TFRC** and **TF** 
  
Found 3207 abstracts in PubMed, retrieved 20.  
 

 What does Google say? 
 PDF only 
| .edu only 

---

**Interactive relationship** (e.g. stimulation, inhibition, etc)

**Stimulatory relationship**- Transferrin  [ **TF** ]  receptor  [ **TFRC** ]  TfR  [ **TFRC** ]  facilitates cellular iron uptake by mediating endocytosis of its ligand, iron loaded transferrin  [ **TF** ] .  Ref: 12881306 BloodBlood,
**Neutral relationship**- Coimmunoprecipitation studies using solubilized cell extracts demonstrated that transferrin  [ **TF** ]   **Tf**  competed with HFE for binding to the transferrin  [ **TF** ]  receptor  [ **TFRC** ]  TfR  [ **TFRC** ]  similar to previous in vitro studies using soluble truncated forms of HFE and the TfR  [ **TFRC** ] .  Ref: 12874382 Proc Natl Acad Sci U S AProc Natl Acad Sci U S A,
- At concentrations of  **Tf**  approaching those found in the blood, no differences in  **Tf**  binding to cells were detected, which is consistent with the lower binding constant of HFE for TfR  [ **TFRC** ]  versus  **Tf** .  Ref: 12874382 Proc Natl Acad Sci U S AProc Natl Acad Sci U S A,

**Non-interactive relationship** (e.g. studied together, co-existance, homology, etc.)

- There was no significant difference in high fluorescence reticulocyte and soluble transferrin  [ **TF** ]  receptor  [ **TFRC** ]  values between the two groups, but a correlation was observed between high fluorescence reticulocytes and soluble transferrin  [ **TF** ]  receptors in iron deficiency anemia, probably due to increased receptor synthesis as a response to decreased iron content in erythrocytes.  Ref: 12870058 Sao Paulo Med J, 2003
- AIM This study compared transferrin  [ **TF** ]  receptor  [ **TFRC** ]  TfR  [ **TFRC** ]  concentrations with iron parameters relative to a child s intake of cow s milk and follow on formula.  Ref: 12856975 Acta Paediatr, 2003
- OBJECTIVE To explore the effect of T 3 on the expression of transferrin  [ **TF** ]  receptor  [ **TFRC** ]  TfR  [ **TFRC** ]  and ferritin Fn in K562 cells and its possible mechanism.  Ref: 12864947 Zhonghua Xue Ye Xue Za Zhi, 2003
- Immunolocalization studies in epithelial cells revealed myo6 was associated with peripherally located vesicles that contained the transferrin  [ **TF** ]  receptor  [ **TFRC** ] .  Ref: 12857860 Mol Biol Cell, 2003
- Rottlerin had no effect on cell surface or total expression of the transferrin  [ **TF** ]  receptor  [ **TFRC** ] , providing evidence that the effect on GLAST cannot be attributed to a non specific internalization degradation of plasma membrane proteins.  Ref: 12859677 J Neurochem, 2003
- Soluble transferrin  [ **TF** ]  receptor  [ **TFRC** ]  and immature reticulocytes are not useful for distinguishing iron deficiency anemia from heterozygous beta thalassemia.  Ref: 12870058 Sao Paulo Med J, 2003
- RESULTS There were no significant differences in hemoglobin, serum transferrin  [ **TF** ]  receptor  [ **TFRC** ] , copper, or zinc among groups.  Ref: 12885712 Am J Clin Nutr, 2003
- Effect of T 3 on the expression of transferrin  [ **TF** ]  receptor  [ **TFRC** ]  and ferritin in K562 cells and its possible mechanism .  Ref: 12864947 Zhonghua Xue Ye Xue Za Zhi, 2003
- However, cells expressing HFE still showed a decrease in  **Tf**  mediated iron uptake at concentrations of  **Tf**  sufficient to dissociate HFE from the TfR  [ **TFRC** ] .  Ref: 12874382 Proc Natl Acad Sci U S AProc Natl Acad Sci U S A, 2003
- An association was found between the concentration of serum soluble transferrin  [ **TF** ]  receptor  [ **TFRC** ]  and the concentration of Mn in whole blood Pearson s r = 0.48.  Ref: 12884407 J Appl ToxicolJ Appl Toxicol, 2003
- The regimen outlined in this brief paper focuses on therapeutically utilizing iron and iron compounds in transferrin  [ **TF** ]  receptor  [ **TFRC** ]  rich tumor cells in ways that inhibit and lyse same.  Ref: 12888304 Med Hypotheses, 2003
- The interface between the two types of regulatory input by insulin is suggested to be the budding of GLUT4 from the transferrin  [ **TF** ]  receptor  [ **TFRC** ]  TfR  [ **TFRC** ]  containing, recycling endosome.  Ref: 12864734 Acta Physiol Scand, 2003
- We further demonstrate that HOCl antagonizes the H2O2 mediated increase in the levels of transferrin  [ **TF** ]  receptor  [ **TFRC** ] , which is a downstream target of IRP1.  Ref: 12888561 J Biol ChemJ Biol Chem, 2003
- IREs are found in the 5 untranslated region UTR of ferritin Ft and mitochondrial aconitase m Aco mRNAs, and in the 3 UTR of transferrin  [ **TF** ]  receptor  [ **TFRC** ]  TfR  [ **TFRC** ]  and divalent metal transporter 1 DMT1 mRNAs.  Ref: 12855587 Blood< MedlineTA>Blood, 2003
- One is the GFP high fraction containing mainly CFU E and proerythroblasts, which coexpress transferrin  [ **TF** ]  receptor  [ **TFRC** ] , while the other is the GFP low transferrin  [ **TF** ]  receptor  [ **TFRC** ]  negative fraction containing BFU E.  Ref: 12893747 BloodBlood, 2003
- Transferrin  [ **TF** ]  receptor  [ **TFRC** ]  1 is differentially required in lymphocyte development.  Ref: 12881306 BloodBlood, 2003
- BACKGROUND Soluble transferrin  [ **TF** ]  receptor  [ **TFRC** ]  sTfR concentration is high in iron deficiency and in conditions of increased erythropoiesis.  Ref: 12867292 Clin Chim ActaClin Chim Acta, 2003
- EXPLY 37 R had no effect on T lymphocyte activation, measured as proliferation as well as expression of early and late cell surface antigens CD69, CD25 IL 2R alpha and CD71  [ **TFRC** ]  transferrin  [ **TF** ]  receptor  [ **TFRC** ]  at the cell membrane.  Ref: 12890427 Int Immunopharmacol, 2003
- Transferrin  [ **TF** ]  receptor  [ **TFRC** ]  in children and its correlation with iron status and types of milk consumption.  Ref: 12856975 Acta Paediatr, 2003
- When fused to unpolarized CD4 or dendritic transferrin  [ **TF** ]  receptor  [ **TFRC** ] , T1 promoted their axonal surface expression.  Ref: 12893943 Science, 2003
- The geometric mean concentration of soluble transferrin  [ **TF** ]  receptor  [ **TFRC** ]  was lower in the exposed subjects than in the controls 2.2 vs 2.6 mg l 1.  Ref: 12884407 J Appl ToxicolJ Appl Toxicol, 2003
- We have previously shown that mouse embryos homozygous for a disrupted transferrin  [ **TF** ]  receptor  [ **TFRC** ]  allele TfR  [ **TFRC** ]  die of anemia before embryonic day 12.5, although most other embryonic tissues appear to be developing normally.  Ref: 12881306 BloodBlood, 2003
- Concentrations of hemoglobin, serum ferritin SF, and serum transferrin  [ **TF** ]  receptor  [ **TFRC** ]  TfR  [ **TFRC** ]  were measured at baseline and after 3 and 6 mo.  Ref: 12885710 Am J Clin Nutr, 2003
- The influence of iron, vitamin B 12, and folate levels on soluble transferrin  [ **TF** ]  receptor  [ **TFRC** ]  concentration in pregnant women.  Ref: 12867292 Clin Chim ActaClin Chim Acta, 2003
- High fluorescence reticulocyte counts and soluble transferrin  [ **TF** ]  receptor  [ **TFRC** ]  levels were determined in iron deficiency anemia patients n = 49 and heterozygous beta thalassemia patients n = 43.  Ref: 12870058 Sao Paulo Med J, 2003
- There were no final differences between groups in hemoglobin, plasma ferritin, or transferrin  [ **TF** ]  receptor  [ **TFRC** ]  concentrations or in the ratio of transferrin  [ **TF** ]  receptors to plasma ferritin after control for initial concentrations.  Ref: 12885707 Am J Clin Nutr, 2003
